# Renaissance of farnesyltransferase inhibitors in cancer

**DOI:** 10.1186/s12967-026-08298-5

**Published:** 2026-05-28

**Authors:** József Tímár, Balázs Hegedűs, Marcell Baranyi

**Affiliations:** 1 Department of Pathology, Forensic and Insurance Medicine, Semmelweis University, Budapest, Hungary; 2 Department of Thoracic Surgery, University Medicine Essen – Ruhrlandklinik, Essen, Germany

**Keywords:** Prenylation, Farnesyltransferase inhibitor, HRAS, RAS inhibitor, Target therapy

## Abstract

**Background:**

Prenylation (geranylgeranylation or farnesylation) plays an important role in the regulation of RAS proteins. This observation led to development of farnesyltransferase inhibitors (FTI) which were extensively tested in clinical studies of various cancer types. Unfortunately, these studies failed and the development of these drugs stalled.

**Main body:**

The renaissance of FTIs started with the approval of lonafarnib in children’s progeria syndromes and continued with the approval of tipifarnib in HRAS-mutant head and neck cancers. Although there are several trials running on various viral infections (HDV, RSV, SARS), their future in oncology is based on the novel preclinical findings that FTIs can potentiate the efficacy of novel KRAS inhibitors or other target therapies such as EGFR- or multikinase inhibitors.

**Conclusions:**

The „second chance” for FTIs is here but the successful clinical implementation of FTIs can only be achieved if the design of the new clinical trials do not repeat mistakes of the past: application of these drugs without predictive markers.

**Supplementary Information:**

The online version contains supplementary material available at 10.1186/s12967-026-08298-5.

## Background: significance of the farnesylation of proteins

Amino acid sequences of the proteins are encoded by the nucleotide sequence in the corresponding genes; however, following translation, they can undergo several distinct further modifications termed post-translational modifications (PTM). One of the most recognized PTMs is phosphorylation, frequently resulting in alterations in the functional activity of the protein. Other PTMs include ubiquitination, regulating proteasomal degradation of the protein; glycosylation, acetylation or methylation with various functional consequences. A unique group of PTMs with a specific function to regulate association with phospholipid membranes is lipid modifications. Lipid modifications include palmitoylation or glycosyl-phosphatidyl-inozitoid (GPI) modification, and prenylation. The latter is one of the most frequent form of lipid modifications, consisting of two major alternatives: CAAX prenyltransferases (FTase and GGTase I), where the takes place at the C-terminal end of the specific protein that contains a CAAX sequence (C marks cysteine, A stands for aliphatic and X for any amino acid) and RAB geranyltransferase (GGTase II) specific for the RAB protein family [1–3]. In this review, CAAX prenylation will be on focus.

**Fig. 1:**
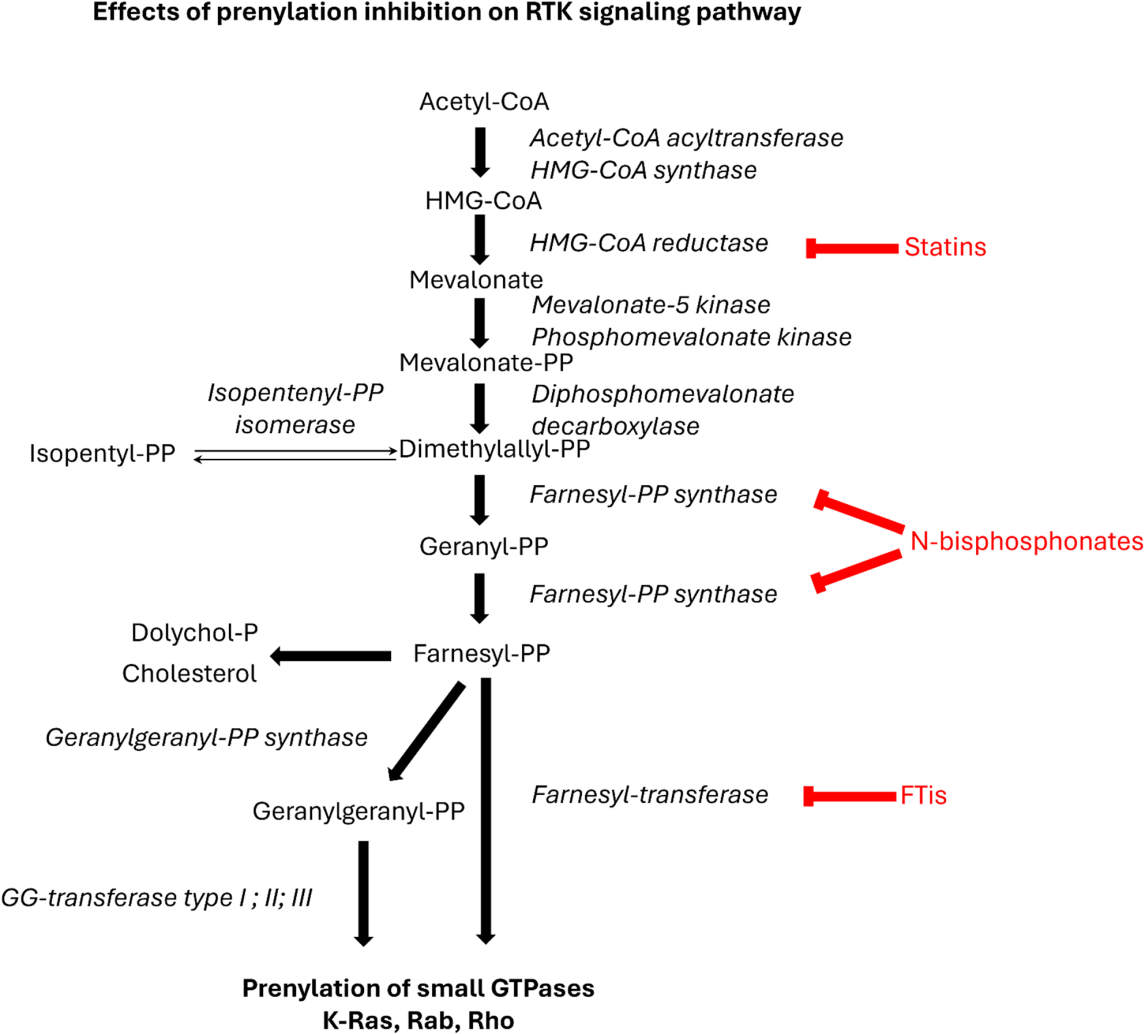
Schematic presentation of the mevalonate synthetic pathway

Terpenoids (isoprenoid- (IPP) or dimethylallyl-pyrophosphates, (DAMPP)) are present in all mammalian cells, synthesized in the mevalonate pathway from acetyl-Coenzyme-A [1–3]. Farnesyl-PP is produced by farnesyl-PP synthase, which can be further transformed into geranyl-geranyl-PP by the geranyl-geranyl-PP synthase (Fig. 1). Accordingly, prenylation of a given protein takes place at the CAAX terminal by covalent linkage of FPP or GGPP by the farnesyltransferase or the geranyl-geranyltransferase (1–4). Notably, distinct variations of the CAAX motif determines if the protein can be farnesylated or geranylated or both. (3,4) Furthermore, recent evidence indicates that numerous proteins with CXXX motif can also be prenylated [5, 6]. Following prenylation, the AAX motif is cleaved by RCE1 and the terminal, prenylated cysteine is methylated by ICMT [3]. Prenylation of a protein can promote lipid membrane association at the cell surface or into the membranes of cytoplasmic organelles such as ER or Golgi [1–7]. 

In mammalian cells, there are a significant number of prenylated proteins involved in key cellular processes likein the regulation of various signal transduction pathways (RAS, RHO), vesicular transport (RAB proteins), kinetochore organization (CENP-E/F) and structural organization within the nucleus, as exemplified by nuclear lamins such as lamin A and lamin B. With regards to cancer, the most important prenylated protein family is the small GTP-ases consisting of more than 150 proteins, including RAS, Rho, Rab, Ran and Arf [1–7]. 

### Significance of prenylation of the RAS proteins

The RAS family has 32 members, but there are three members which have crucial oncogenic role: KRAS, NRAS and HRAS [8]. As it was mentioned before, prenylation takes place at the C-terminal CAAX domain of these proteins. Due to variation in the CAAX sequence, while KRAS and NRAS can be prenylated both by farnesylation and geranyl-geranylation, HRAS can only be farnesylated [2]. Notably, the lipid modification of the two main splice variants KRAS, 4 A and 4B, is different as membrane association of KRAS4a (similarly to NRAS and HRAS) is further regulated by the reversible palmitoylation, this process does not take place in the case of KRAS4b. Instead, the membrane localization of this latter splice variant is promoted by C-terminal polybasic sequences [2]. Prenylation of RAS proteins fosters membrane localization and the possibility to interact with other membrane-bound proteins such as PI3K and GEF/GAP proteins. The GEF interaction activates RAS by GDP/GTP exchange, while GAPs inactivate them by enhancing their GTPase activity. Activation of RAS promotes RAF binding and activates the MEK/ERK pathway, while on the other hand, interaction with PI3K would initiate AKT signaling. Prenylation of RAS also facilitates dimerization or nanoclustering, which promotes RAF/MEK/ERK signaling activation but also facilitates calmodulin binding, which is necessary for PI3K activation [3]. 

RAS mutations are among the most common genetic alterations in human cancer [6]. KRAS mutation is most prevalent in lung, colorectal and pancreatic cancers. NRAS mutation occurs in melanoma, thyroid cancer or colorectal cancer while HRAS mutations are often found in head and neck or urinary bladder cancer but to a lower frequency in various other cancer types [8]. As mutant RAS proteins also rely on prenylation for membrane localization, farnesyltransferase enzymes emerged as a potential target to inhibit oncogenic RAS functions and to treat RAS mutant tumors.

## Indirect and direct farnesyltransferase inhibitiors (FTI)

### Statins

Statins are the main cholesterol-lowering drugs which are HMG Coenzyme-A reductase inhibitors [9]. Statins inhibit the production of mevalonate, resulting in decreased production of IPP/DAMPP, which would result in decreased prenylation substrate production. Inhibition of the mevalonate pathway has pleiotropic effects where the prenylation inhibition cannot be clearly separated from the inhibition of cholesterol biosynthesis. To inhibit prenylation, statins must reach high plasma concentrations, where side effects are more prevalent. Analysis of the DEPMAP database indicated that statins in vitro have antiproliferative effects on KRAS wild-type cancer cell lines [7]. 

### N-bisphosphonates

N-bisphosphonates (alendronate, ibandronate, neridronate, olpadronate, pamidronate, risedronate, and zoledronate) are key drugs to treat osteoporosis and bone metastatic cancers [10, 11]. N-bisphosphonates are inhibitors of farnesyl-PP synthase and to a lesser extent geranyl-PP synthase as well, resulting in inhibition of prenylation. On the other hand, all classes of bisphosphonates selectively accumulate in mineralized bone. However, the newly developed lipophilic-N-bisphosphonates have a broader tissue distribution [12]. Analysis of the DEPMAP database demonstrated that N-bisphosphonates have more prominent antitumoral effect on KRAS mutant cancer cell lines, although this effect may vary depending on tissue of origin [7]. 

### Direct farnesyltransferase inhibitors

Farnesyltransferase inhibitors are specific small molecular inhibitors of the farnesyltransferases [13]. The best known drug of this class is tipifarnib (Suppl. Figure 1), which is a quinolinon-derivative and lonafarnib (Suppl. Figure 1), which is a tricyclic carboxamide derivative. Further inhibitors include BMS-214,662 (Suppl. Figure 1), which is a benzodiazepine derivative while L-778,123 (Suppl. Figure 1) is an oxipiperazin-imidazole, which is a dual farnesyl- and geranyl-transferase inhibitor. More recently, a so-called second generation FTI, KO2806 (Suppl. Figure 1), was developed, which is currently under clinical investigation [14]. The unique GGTI-2418 (Suppl. Figure 1) is a peptidomimetic with specific geranyltransferase specificity. Furthermore, dual FT/GT inhibitor (FGTI-2734, Suppl. Figure 1) was also developed early on [15]. Although there are several other FTIs and GTIs developed, most of them never reached clinical application [13]. Analysis of the DEPMAP database demonstrated that FTIs have a strong antitumoral effect on cancer cell lines. Specifically in the case of colorectal cancer KRAS wild-type cell lines were more sensitive, while in the case of lung cancer cell lines, the opposite effect could be observed [7]. 

## Clinical studies with farnesyltransferase inhibitors in the past

There has been numerous clinical studies performed with direct farnesyltransferase inhibitors on various cancer patient cohorts in the beginning of the 21st century. The majority of the studies were monotherapy trials (Table 1.), but there have been several trials on combination with chemotherapeutic agents. (Table 2.) It is typical that most of the studies never reached phase-II due to ineffectiveness. From the 70 FTI trials, only four reached phase-III: a tipifarnib trial on colorectal cancer, another tipifarnib monotherapy trial on AML, a chemotherapy combination of tipifarnib on pancreatic cancer and a chemotherapy combination of lonafarnib on NSCLC. Besides these two FTIs, BMS-214,662 and L-778,123 have been also tested clinically without success [13, 16]. Unfortunately, clinical data have not been evaluated according to the RAS mutation status of the cancers. Nevertheless, among patients with objective responses, types of cancers with KRAS wild-type status were predominant. Lastly, there was another trial with tipifarnib in combination with chemotherapy in triple-negative breast cancer, again without noticable clinical activity [17]. 

**Table 1:** Failed clinical trials with farnesyltransferase inhibitor monotherapy

cancer types	Tipifarnib (*n*)	Lonafarnib (*n*)	BMS-214,662 (*n*)
solid tumors	4 (p1)	5 (p1/2)	0
NSCLC	1 (p1)	1 (p1)	0
SCLC	1 (p2)	0	0
**colorectal cancer**	**2 (p2/3)**	1 (p2)	0
breast cancer	1 (p2)	0	0
pancreatic cancer	2 (p2)	0	0
bladder cancer	1 (p2)	1 (p2)	0
glioblastoma	1 (p2)	0	0
**AML**	**3 (p2/3)**	1 (p2)	0
AL	1 (p1)	0	1 (p1)
CML	1 (p2)	1 (p1)	0
MyM	1 (p2)	0	0
total (30)	19	10	1

AL= acute leukemia, CML= chronic myeloid leukemia, MyM=myeloma multiplex, NSCLC = non-small cell lung cancer, p= phase (1/2/3) clinical investigation, SCLC= small cell lung cancer

**Table 2:** Failed clinical trials of farnesyltransferase inhibitors in combination with chemotherapy

cancer type	Tipifarnib (*n*)	Lonafarnib (*n*)	BMS-214,662 (*n*)
solid tumors	6 (p1)	2 (p1)	4 (p1)
**NSCLC**	0	**1 (p3)**	0
breast cancer	4 (p1/2)	0	0
**pancreatic cancer**	**1 (p3)**	0	0
bladder cancer	0	1 (p2)	0
AML	2 (p1/2)	0	0
CML	1 (p1)	1 (p1)	0
total (23)	14	5	4

AML= acute myeloid leukemia, CML= chronic myeloid leukemia, NSCLC = non-small cell lung cancer, p= phase (1/2/3) clinical investigation

A noteworthy attempt was made in a trial where tipifarnib monotherapy was tested on neurofibromatosis (an NF1 mutant tumor) without success [18]. On the other hand, tipifarnib monotherapy was tested on peripheral T-cell lymphoma, where RhoA and IDH2 mutant cases showed some clinical responses [19]. Tipifarnib was also tested in combination with various targeted therapeutic agents. One of these attempts was the combination of tipifarnib with imatinib in BCR-ABL-positive CML. (13) Another combination was a trial with the EGFR inhibitor erlotinib in various cancer types [20]. The trial combining tipifarnib with the multikinase inhibitor sorafenib in glioblastoma also failed [21]. 

Collectively, it can be stated that the failures of FTIs in these clinical trials can be explained primarily by the lack of clinical effectivity but not by intolerable toxicity. The negative clinical performance of FTis can be partially explained by the discovered capability of KRAS and NRAS for alternative prenylation [22]. On the other hand, these trials of a targeted agent have been designed without predictive biomarkers. These negative results downgraded of FTIs as anticancer agents in an era when the search for inhibitors against the non-druggable RAS was more and more in the focus of preclinical research.

## Renaissance of the farnesyltransferase inhibitors

### Non-cancerous diseases

The exploration of FTIs in non-malignant diseases started with a real success. In children, there are two progeria syndromes (PG), a disease characterized by accelerated aging: the Hutchinson-Gilford-PS (HG-PS) and the progeroid laminopathy (PL) [23]. HG-PS is an autosomal dominant disease of PS caused by LMNA mutation. As a consequence, premature lamin-A accumulates in the nucleus in prenylated form. In the other form, PL, mutations can occur in LMNA or ZMPSTE24 [23]. Clinical consequences of these genetic alterations are progeria of children and the occurrence of stroke or aggressive cardiovascular diseases. As a consequence, the median survival of the affected children is ~ 15 years. There have been two clinical trials on HG-PG where the FTI lonafarnib was tested for clinical efficacy. In the first part, lonafarnib was applied to HGPG patients orally at a dose of 115 mg/kg/m^2^ for 6 months followed by an increased dose of 150 mg for two and a half years. In the second phase of the study, lonafarnib treatment with the higher dose was applied for 8 years in HGPG patients. In the second phase of the clinical investigation, a lonafarnib-naive HGPG control group was also established. In the entire study, 62 Lonafarnib-treated and 81 control HGPG patients have been enrolled and the overall survival was analyzed. In the 3-year lonafarnib treatment group, OS was increased by 2.6 years and the advantage is maintained in the 11-year treatment group compared to the control group. Following 5-year of treatment, the chance for survival was 79% as compared to the 57% of the control group. At 10 years of treatment, OS was 44% compared to the 17% of the control group [23, 24]. Based on these results FDA approved lonafarnib therapy of HGPG patients in 2020, marking the first clinical approval of a farnesyl-transferase inhibitor [24]. 

The hepatitis D-viral antigen is a prenylated protein playing an important role in the viral infection. HDV is responsible for a significant portion of viral hepatitis, and there is no available vaccine developed for the prevention of HDV infections yet [25]. Lonafarnib was tested in HDV hepatitis patients using orally twice-daily at the doses of 100 and 200 mg/kg/m^2^. Analysis of the HDV in blood indicated that the treatment decreased viral particles, and the higher dose was more effective in this respect [26]. In another phase II clinical trial, lonafarnib was combined with ritonavir and PEG-IFNα in chronic HDV infected patients. Lonafarnib in combination with ritonavir effectively eliminated HDV from the blood both at full and lower doses, especially when the latter was combined with PEG-IFNα [27]. In a preclinical study, lonafarnib was also effective to prevent RSV infection by the inactivation of the RSV fusion protein-F [28]. An AI-based search for potential antiviral COV-2 omicron drugs identified FT as a potential drug target. In the preclinical study, both tipifarnib and lonafarnib were able to prevent COV-2 infection and the development of cytopathic effects [29]. FT of the Plasmodium falciparum was also identified as a possible drug target, suggesting FTIs as novel class of antimalaria drugs [30]. FTIs (tipifarnib and lonafarnib) have been found effective also to treat antibiotic resistant bacterial infections such as MRSA [31]. 

### Antitumoral effects of the bisphosphonates

The revolution of the treatment of bone-metastatic cancers started with the anti-osteoclast agents, bisphosphonates, accumulating in the bone mineral matrix. As it was mentioned before, the second-generation N-bisphosphonates are prenylation inhibitors [10, 11] and it was suggested that they may have a direct anti-tumoral effect [32]. In preclinical models, it was shown that zoledronate has antitumoral effect in KRAS-wild-type lung cancer cell lines exclusively [33]. The novel lipophilic bisphosphonate BPH1222 [10] was shown to inhibit KRAS mutant colorectal cancer cells [34]. Analysis of a bisphosphonate-treated NSLC cohort demonstrated an OS benefit in the KRAS wild-type patients, (35) which was in line with previous preclinical data [33]. 

### Targeted therapy of HRAS mutant cancers

**Fig. 2:**
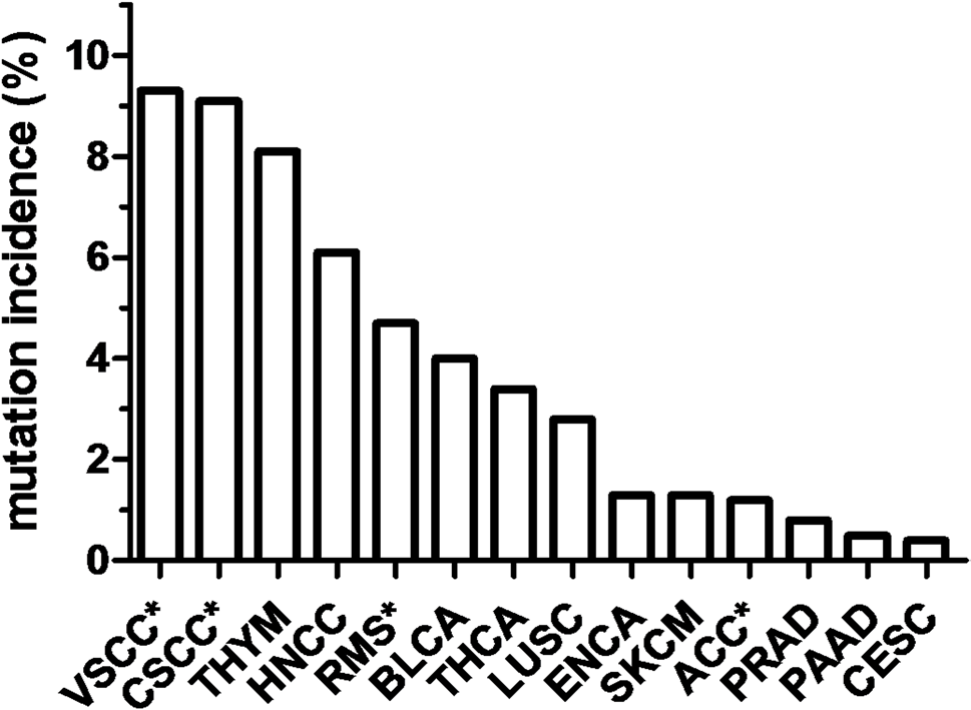
Incidence of HRAS mutations in human cancers (data from TCGA except for entities marked with asterisk, refs. [34–37]). ACC – salivary gland adenocystic carcinoma, BLCA – bladder urothelial carcinoma, CESC – cervical squamous cell carcinoma, CSCC – cutaneous squamous cell carcinoma, ENCA – endometrial cancer, HNCC – head and neck squamous cell carcinoma, LUSC – lung squamous cell carcinoma, PAAD – pancreatic adenocarcinoma, PRAD – prostate adenocarcinoma, RMS – rhabdomyosarcoma, SKCM – skin cutaneous melanoma, THCA – thyroid cancer, THYM – thymoma, VSCC –vulvar squamous carcinoma

Analysis of the TCGA database indicates that > 5% frequency of HRAS mutation can be found in various types of squamous cancers (cervical, vulvar, head and neck, lung) or in urinary bladder cancer. However, HRAS mutation occasionally occurs in various adenocarcinomas (pancreatic, thyroid, ovarian or endometrial), in glioblastoma or in childhood pre-B-ALL. (Fig. 2, refs. [36–39]). Collectively, it can be stated that HRAS mutant cancers are not rare and affect several types of cancers, where at the moment, there is no effective target therapy.

Analysis of a large HNSCC cohort indicated that HRAS mutation is independent from HPV infection but is more frequent in HPV-negative cancers. It is of note that in this cancer type, the most frequent mutation type is exon2-G12S or G13, but exon3Q61 is also prevalent. Co-occuring mutations with HRAS are CASP8, TERT, and NOTCH1 in HNCC. Unlike in KRAS mutant NSCLC, KEAP1 and STK11 mutations are absent in HRAS mutant HNSCC [40]. In the KO-TIP001 phase-II trial, 20 HRAS mutant advanced HNSCC patients were enrolled who had progressed on a first line therapy. The uniqueness of this trial is that this was the first targeted therapy clinical trial where the frequency of the mutation in the tumor was a stratification factor, since only those patients were eligible whose tumors contained HRAS mutation at a higher than 20% VAF level. This meant that only those tumors were eligible where HRAS mutation was clonal (assuming a heterozygous mutation status). Patients have been treated orally with tipifarnib twice a daily oral dose of 600 or 900 mg in 28-day intervals. The overall response rate in the treated population was 55%, the PFS was 5.6 months as compared to the 3.6 months of the various previous treatments, while median OS was found to be 15.4 months. Concerning side effects, 37% of the patients developed anemia, and in 13% lymphopenia was detected. It is important that all the treated patients experienced benefit from tipifarnib treatment since the partial response (PR) rate was 11/20 and the stable disease (SD) rate was 9/20. It is also important that the clinical efficacy of tipifarnib was independent from the type of HRAS mutation [41]. Based on these data FDA approved tipifarnib for the treatment of HRAS mutant advanced HNSCC. Recently, Tipifarnib has also been tested in a phase II clinical trial for the treatment of HRAS wild-type but PI3KCA mutant HNSCC in combination with alpelisib [42]. 

HRAS mutation rate in urothelial cancer is also relatively high. (Fig. 2) Data indicates that HRAS mutations are more prevalent in smoking associated cases, while in the non-smoker cases KRAS and NRAS mutations are more frequent [43]. Identification of mutant HRAS in the urine in case of hematuria is a diagnostic help to discriminate malignant cases [44]. Furthermore, local recurrence of the malignant tumor can be sensitively diagnosed by mutant HRAS detection in the urine [45]. Germline HRAS mutation occurs in Costello syndrome, where epithelial lesions (benign and malignant) are frequent in the urinary bladder. In these patients, although the majority of the lesions are benign, around 20% of the cases proved to be malignant [46]. A phase II clinical trial was performed in 2005 with Tipifarnib monotherapy on metastatic bladder cancer, but the PR rate was disappointingly low [47]. Learning from the experience with HRAS mutation-associated bladder cancer, a new trial was initiated on metastatic bladder cancer based on the selection of patients with HRAS and STK11 mutations. Twenty-one HRAS mutant patients have been **e**nrolled who were treated orally with tipifarnib monotherapy at a dose of 793 mg/week. The ORR was 33% and the SD rate was 19% therefore, the DCR was found to be 52%. It is of note that HRAS wild-type patients did not respond to therapy. As a consequence, the PFS in the HRAS mutant group was significantly longer as compared to the HRAS wild-type group: 5.1 versus 0.8 month [48]. In this trial FTI monotherapy missed pivotal thresholds and the rate of early discontinuation was very high thought to be related to adverse events. It is interesting since in the HNSCC trial the dose of tipifarnib was very similar but did not cause so frequent side effects [47]. The conclusion of the metastatic bladder cancer trial was that the monotherapy is not effective enough and it has to be combined with other modalities such as immunotherapy.

Tipifarnib monotherapy was also tested in a small cohort of HRAS mutant salivary gland carcinoma patients. While only one response was detected, the SD ratio was 58% and the PFS was 7 months while the OS was 22.4 months, suggesting clinical activity of the treatment [49]. 

## The future of farnesyltransferase inhibitors

### FTI monotherapy

HRAS mutant cancers are numerous, and FTIs have been clinically tested only in three of them: head and neck, bladder, and salivary gland cancers. It is expected that FTIs would be evaluated in several of HRAS mutant cancer types, such as other squamous cell carcinomas, thyroid cancer or the childhood tumor rhabdomyosarcoma. It is of note that a new tumor-agnostic clinical trial with the FTI, KO-2806, is on the way in various cancers that carry H/K/NRAS mutations [50]. 

Furthermore, recent preclinical evidence suggests that cellular models with increased expression of the beta subunit of the FTase complex (FNTB) may indicate NRAS sensitivity to FTI treatment [5]. Since in skin melanoma the second most frequent mutation is in NRAS, farnesyl-transferase inhibition may be a promising candidate for the targeted therapy.

Uveal melanoma is characterized by GNAQ or GNA11 mutations. Recently it was discovered that these genetic alterations induce overexpression of the prenylated protein INPP5A [51]. It was demonstrated in a preclinical study that the FTI monotherapy (tipifarnib or lonafarnib) of uveal melanoma cell lines carrying the above mentioned gene mutations and overexpressing INPP5A, has a significant anticancer effects [5]. 

It could be very important to discover resistance mechanisms for FTI monotherapy of HRAS mutant cancers. In a large in-silico and preclinical study it was discovered that tipifarnib monotherapy resistance was associated with distinct co-mutations of various genes, such as PTEN, NF1, BRAF class 3, and PIK3CA [52] while in another analysis, the GNAS mutation was responsible for tipifarnib resistance of thyroid cancer models [53]. 

### Combination with KRAS inhibitors

KRAS G12C inhibitors have been the first pioneers of mutation-specific RAS inhibitors, which have reached the clinic: sotorasib, adagrasib and divarasib. Two of them, sotorasib and adagrasib have already been approved in G12C mutant lung cancer [54]. Of note, there are two approved KRAS G12C inhibitors developed and approved in China (fulzerasib and garsorasib). However, the effectiveness of these G12C inhibitors proved to be relatively limited due to emerging acquired resistance. Accordingly, recent development focus is on defining clinically effective combinations involving SOS1 or SHP2 inhibitors, immunotherapies, RAS signaling pathway inhibitors such as MEK- or PI3CA-inhibitors. (54) Preclinical studies indicated that treatment with KRAS G12C inhibitors induced rewiring of the KRAS-related signaling, which is abrogated by the addition of farnesyl-transferase inhibitors that block the compensatory activation of two farnesylated proteins, HRAS and RHEB small GTPases [55, 56]. Interestingly, upon RHEB activation mTORC1 emerged as important mediator of mutant RAS inhibitor resistance [57]. Experimental data indicated that the combination of KRAS G12C inhibitors with various FTIs resulted in synergistic antitumoral effects in various human cancer cell lines and in xenograft models [55–57]. The concept is further corroborated by the preclinical data that a dual FTI/GGTI FGTI-2734 can also synergistically increase the efficacy of the G12C inhibitor sotorasib in human lung cancer cell lines, xenografts and PDX models [58]. 

The most frequent mutant form of KRAS in human cancers is G12D with highest incidence in pancreatic and colorectal cancers [8]. The first KRASG12D inhibitor reaching clinical testing is MRTX1133 [59]. Preclinical data demonstrated that the combination of MRTX1133 with various FTIs has also synergistic antitumoral effects in various human cancer cell lines [60]. Notably, this combination was effective in those cell lines which were relatively resistant to G12D inhibitor monotherapy [60]. Furthermore, in a more recent study G12D mutant human pancreatic cancer cell lines and PDX tumors were treated with MTX1133 and FGTI-2734, where the combination was much more effective than the G12D inhibitor monotherapy [61]. 

The most frequent KRAS mutation following G12D and G12C is G12V, primarily occurring in the same tumor types as G12D. At this point, there is no mutation specific KRAS inhibitor available against this mutation. It is a novel strategy to develop pan-RAS inhibitors that do not have allele-specificity [62]. Experimental data indicate that several of those pan-RAS inhibitors are active on G12V mutant cancer cell lines [62]. Preclinical data suggest that combination of various FTIs with pan-RAS inhibitor has a similar synergistic antitumoral effect as it was observed in the case of G12C or G12D inhibitors [63]. 

### Other possible combinations with targeted therapy

Preclinical data suggested a synergistic antitumoral effects of EGFR TK inhibitor erlotinib and tipifarnib in various human cancer cell lines. Based on these observations, a phase I study was initiated to find an effective and tolerable combination regimen. Twenty-seven patients have been enrolled, 15 for dose escalation and 12 for dose expansion. MTD was 150 mg/day erlotinib and 300 mg/ twice daily for tipifarnib. PR was 7.4% while SD was 37% in this heterogeneous patient population [20]. More recently, FT was found to play a key role in driving various target therapy resistances in human NSCLC models. Interestingly, this mechanism was observed in T790M mutant lung cancer model where resistance to the third generation EGFR TKI, osimertinib is relevant clinically. In human lung cancer cell lines and PDX models combination of osimertinib with tipifarnib increased the efficacy of the target therapy which effect was solely dependent on the FT activity [64]. 

In HRAS wt HNSCC PIK3CA mutations are relatively frequent, where HRAS was found to be overexpressed. In preclinical models, the combination of tipifarnib with PIK3 inhibitor alpelisib had a strong antitumoral effect, and a clinical trial is on the way to test this combination [65]. Meanwhile, there are preclinical data that in HRAS mutant cancers with PIK3CA mutations tipifarnib-combinations with various PIK3 inhibitors were ineffective, but the combination with MEK inhibitor was effective [5]. 

Antiangiogenic therapy of RCC is based on the use of various VEGFR TK inhibitors. However, the clinical efficacy is not high and resistance develops relatively early. Cabozantinib is a multikinase inhibitor with VEGFR2 inhibitory profile and was found to be relatively ineffective clinically in case of RCC with progression after treatment by first generation angiogenesis inhibitors such as axitinib or levantinib. Using in vivo xenograft models, cabozantinib was combined with the next-generation FTI, KO-2806 in human angiogenesis-inhibitor-resistant RCC models. Data indicated that KO-2806 promoted the antitumoral effects of cabozantinib, which was due to a more pronounced antiangiogenic effect. Furthermore, combination of other VEGFR TKIs such as axitinib or levantinib with KO-2806 was significantly more effective in vivo in preclinical models of human RCC [66]. 

### ALK mutant neuroblastoma

A proportion of neuroblastomas is characterized by ALK mutation/amplification and these tumors are treated by ALK-TKIs. It was found that in ALK-altered neuroblastomas the NRAS regulator, miR1304-5p, is lost. Interestingly, in such neuroblastoma model combination of ALK-TKI with lonafarnib resulted in synergistic antitumoral effects in vitro and in PDX tumors [67]. 

## Conclusions

FTIs have a long history of basic research and clinical development. However, they have demonstrated that successful clinical application of a drug class requires careful selection of cancer types as well as identification of the key predictive markers. It is interesting that this drug class re-emerged after historical failures, which is due to the pivotal role of farnesylated proteins in cancer biology. However, the successful clinical implementation can only be achieved if the design of the new clinical trials does not repeat mistakes of the past: application of these drugs without predictive markers. The „second chance” for FTIs is here since strong preclinical data suggest their potency as monotherapy or promising partners for combination with targeted agents.

## Supplementary Information

Below is the link to the electronic supplementary material.

Supplementary Material 1

## Data Availability

Not applicable.

## Abbreviations

AML: Acut myeloid leukemia
CML: Chronic myelogenous leukemia
DAMPP: Dimethylallyl-pyrophosphate
CR: Disease control rate
DEPMAP: Dependency map project
DCTR: Disease control rate
FPP: Farnesyl-pyrophosphate
FT: Farnesyltransferase
FTI: FI-inhibitor
GAP: GTP-ase activating protein
GEF: Guanine nucleotide exchange factor
GGPP: Geranyl-geranyl-pyrophosphate
GPCR: G-protein coupled receptor
GTI: Geranyl-geranyltrasferase inhibitor
HDV: Hepatitis-D virus
HNCC: Head and neck cancer
HPV: Human papilloma virus
IPP: Isoprenoid pyrophosphate
MRSA: Methycillin resistant staphylococcus aureus
MTD: Maximal tolerated dose
NSCLC: Non-small cell lung cancer
ORR: Overal response rate
OS: Overal survival
PDX: Patient-derived xenograft
PFS: Progression-free survival
PG: Progeria syndrome
PR: Partial response
PTM: Post-translational modification
RCC: Renal cell cancer
SD: Stable disease
TKi: Tyrosine kinase inhibitor
VAF: Variant allele frequency
