# Supplementary figures and images for: Renaissance of farnesyltransferase inhibitors in cancer
Source: J Transl Med. 2026 May 28;24:974. doi: 10.1186/s12967-026-08298-5 (PMC13422314; doi:10.1186/s12967-026-08298-5)

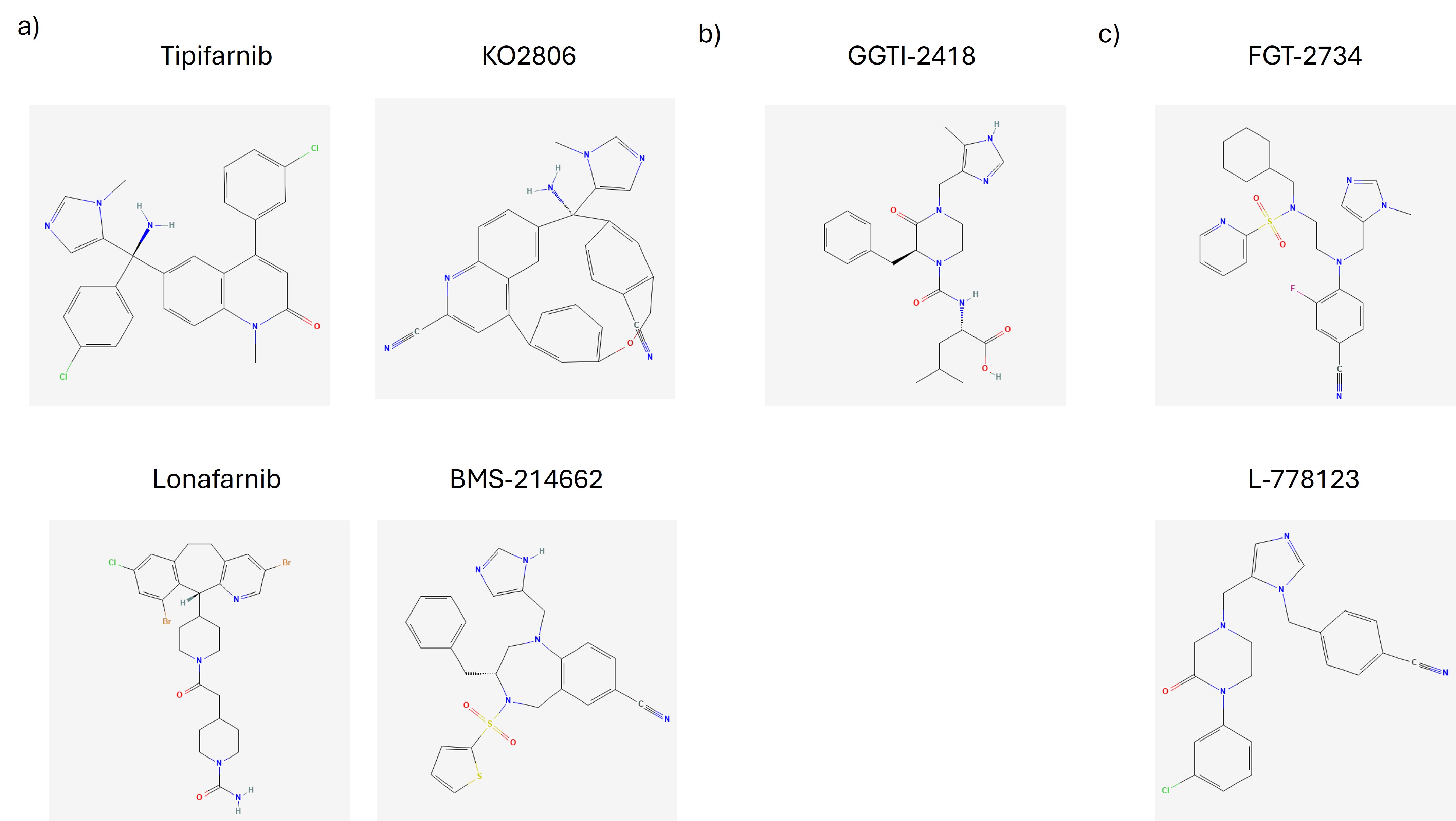

Supplement: Supplementary file 1 — Supplementary Material 1 [file 12967_2026_8298_MOESM1_ESM.jpg]
